# Supplementary material for: Control of Dielectric, Mechanical, and Thermal Properties of a Polymer Composite Based on ABS Using CoB Nanoparticles
Source: Polymers (Basel). 2024 Dec 27;17(1):38. doi: 10.3390/polym17010038 (PMC12974218; doi:10.3390/polym17010038)
Supplement: Supplementary file 1 [file polymers-17-00038-s001.zip › polymers-3378528-supplementary.pdf]

Electronic supporting information for article:

Control of Dielectric, Mechanical, and Thermal Properties of a Polymer Composite Based on ABS Using CoB Nanoparticles

Artur Khannanov [1,*], BAnastasia Burmatova [1], Dinar Balkaev [1], Anastasia Rossova [1] , Konstantin Zimin [1], Airat Kiiamov [2] , Mikhail Cherosov [2], Ivan Lounev [2] and Marianna Kutyreva [1]

1 A.M. Butlerov Chemical Institute, Kazan Federal University, Kazan 420008, Russia;
anaeburmatova@kpfu.ru (A.B.); dinar.balkaev@yandex.ru (D.B.); anastasia.rossova@yandex.ru (A.R.);
kostzim@list.ru (K.Z.); mkutyreva@mail.ru (M.K.)
2 Institute of Physics, Kazan Federal University, Kazan 420008, Russia; ajrat.kiyamov@kpfu.ru (A.K.);
miacherosov@kpfu.ru (M.C.); lounev75@mail.ru (I.L.)
* Correspondence: arthann@gmail.com

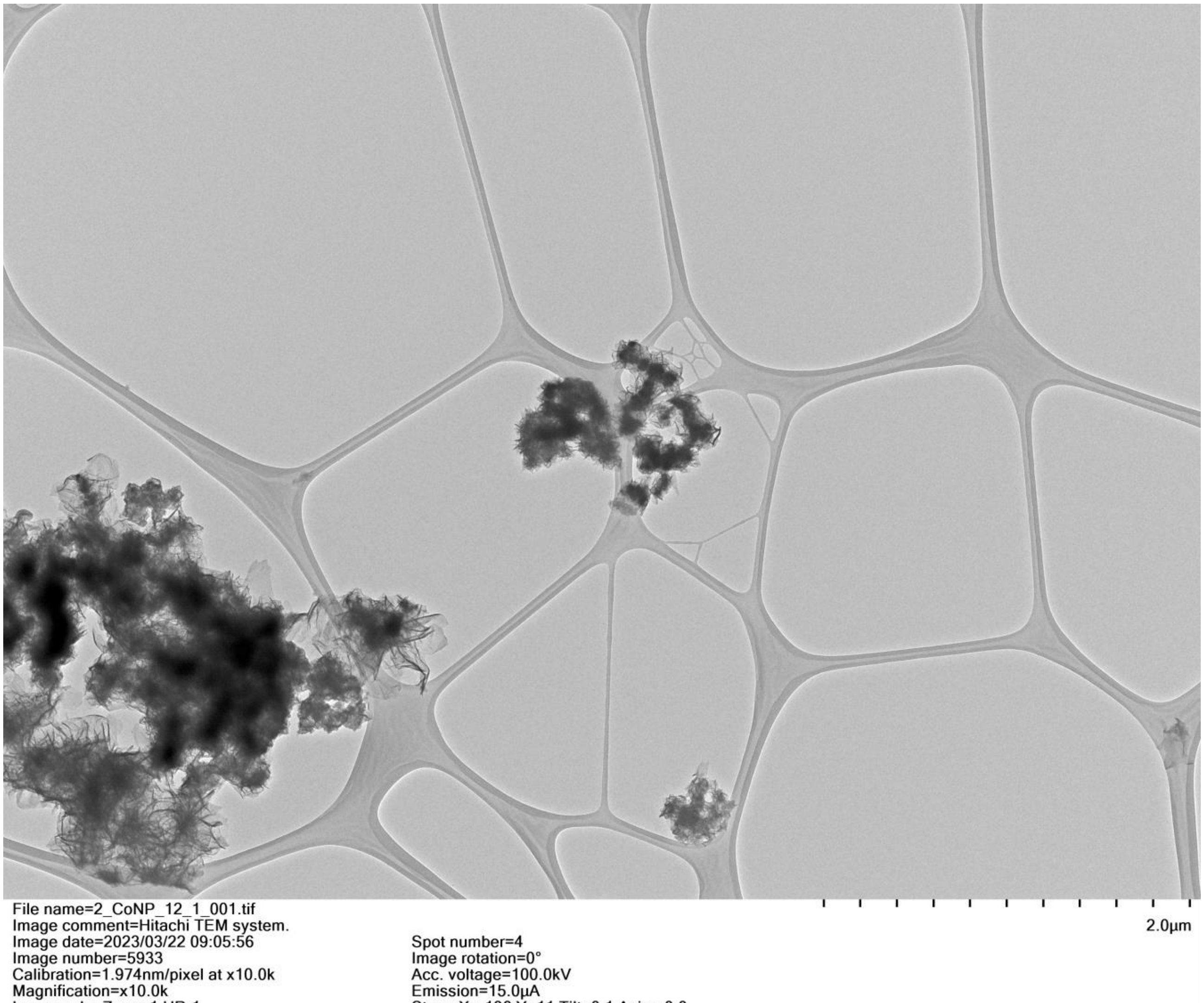

Figure S1. Tem image PEG/CoB-NP

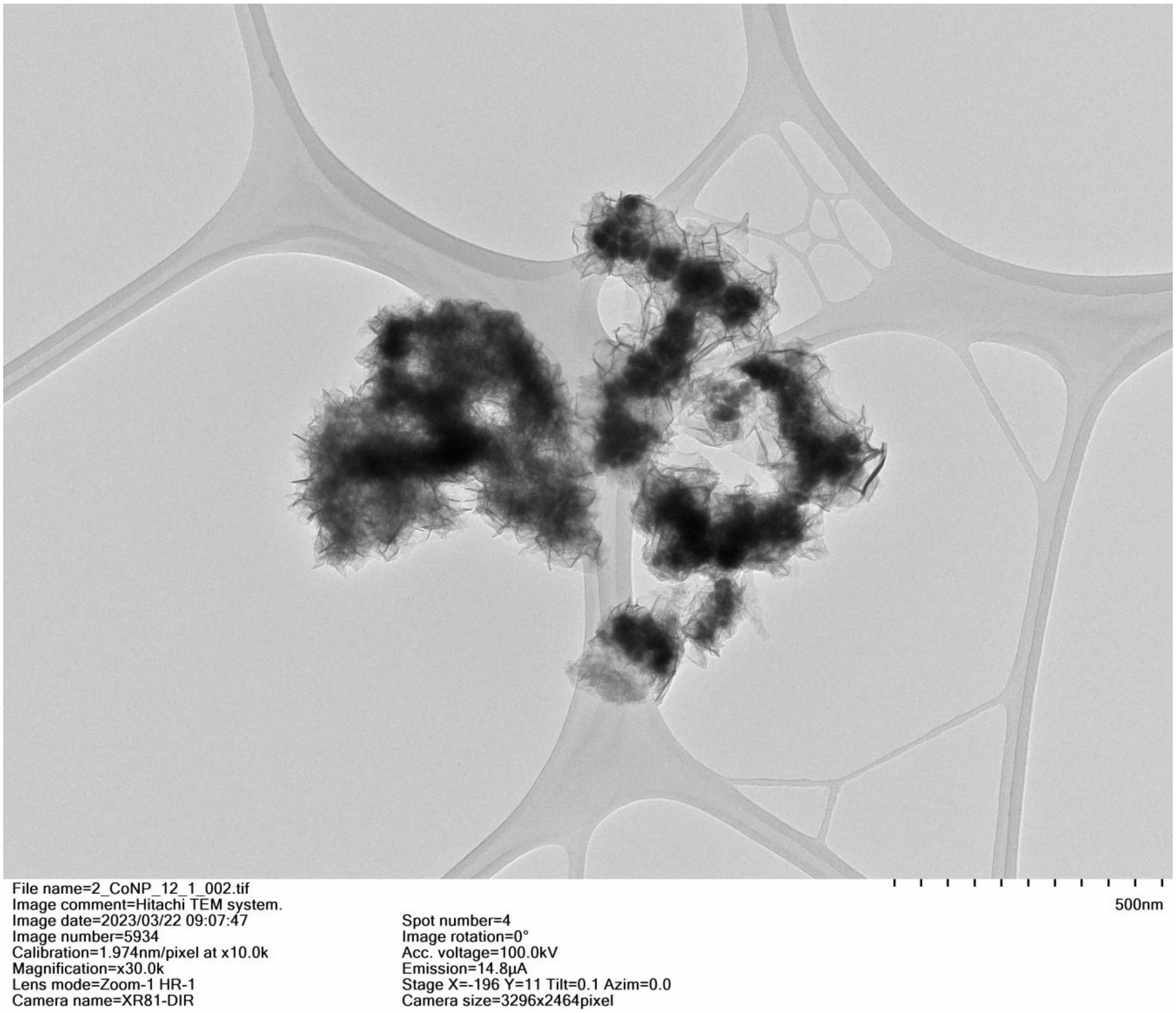

Figure S2. Tem image PEG/CoB-NP

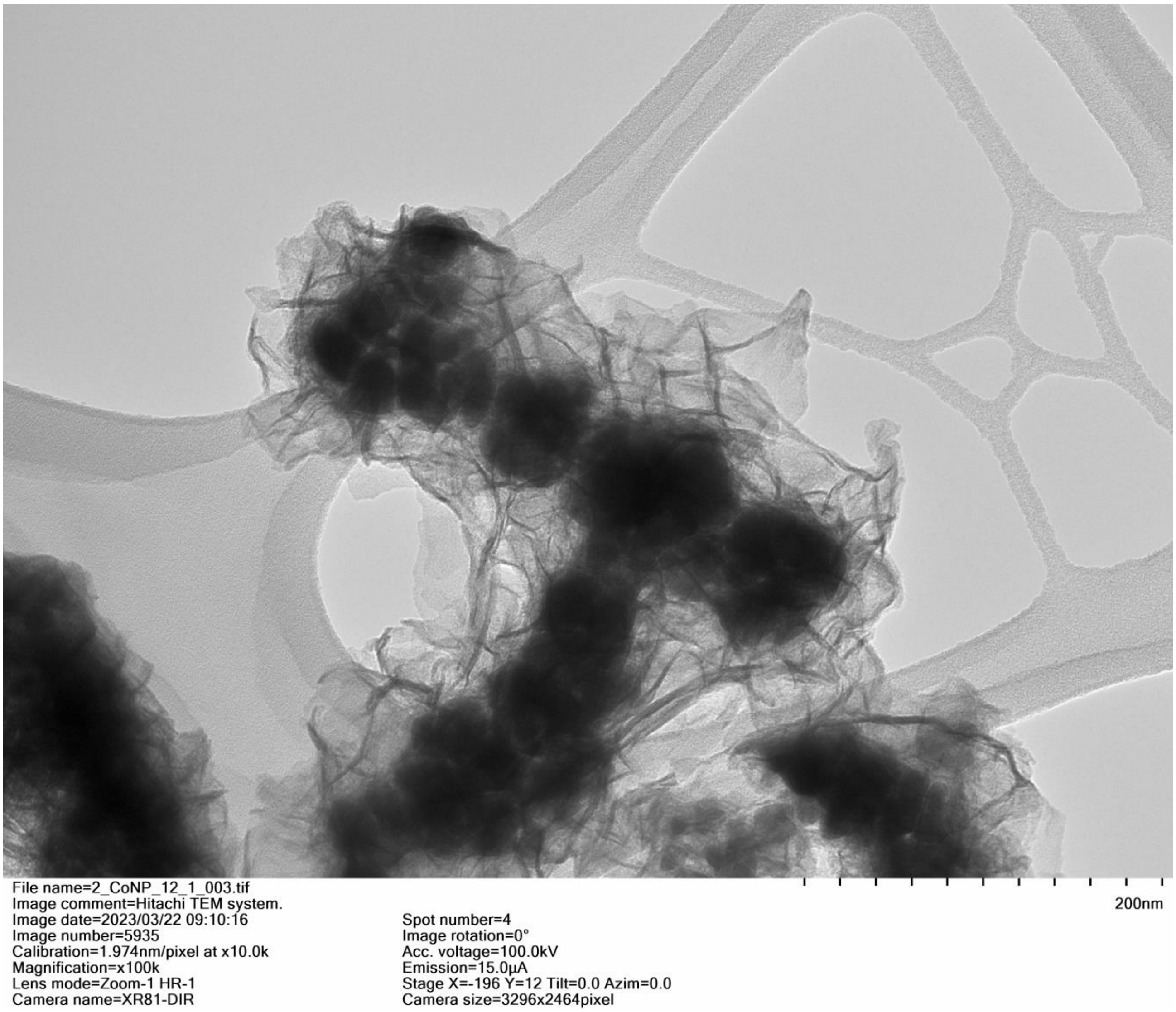

Figure S3. Tem image PEG/CoB-NP

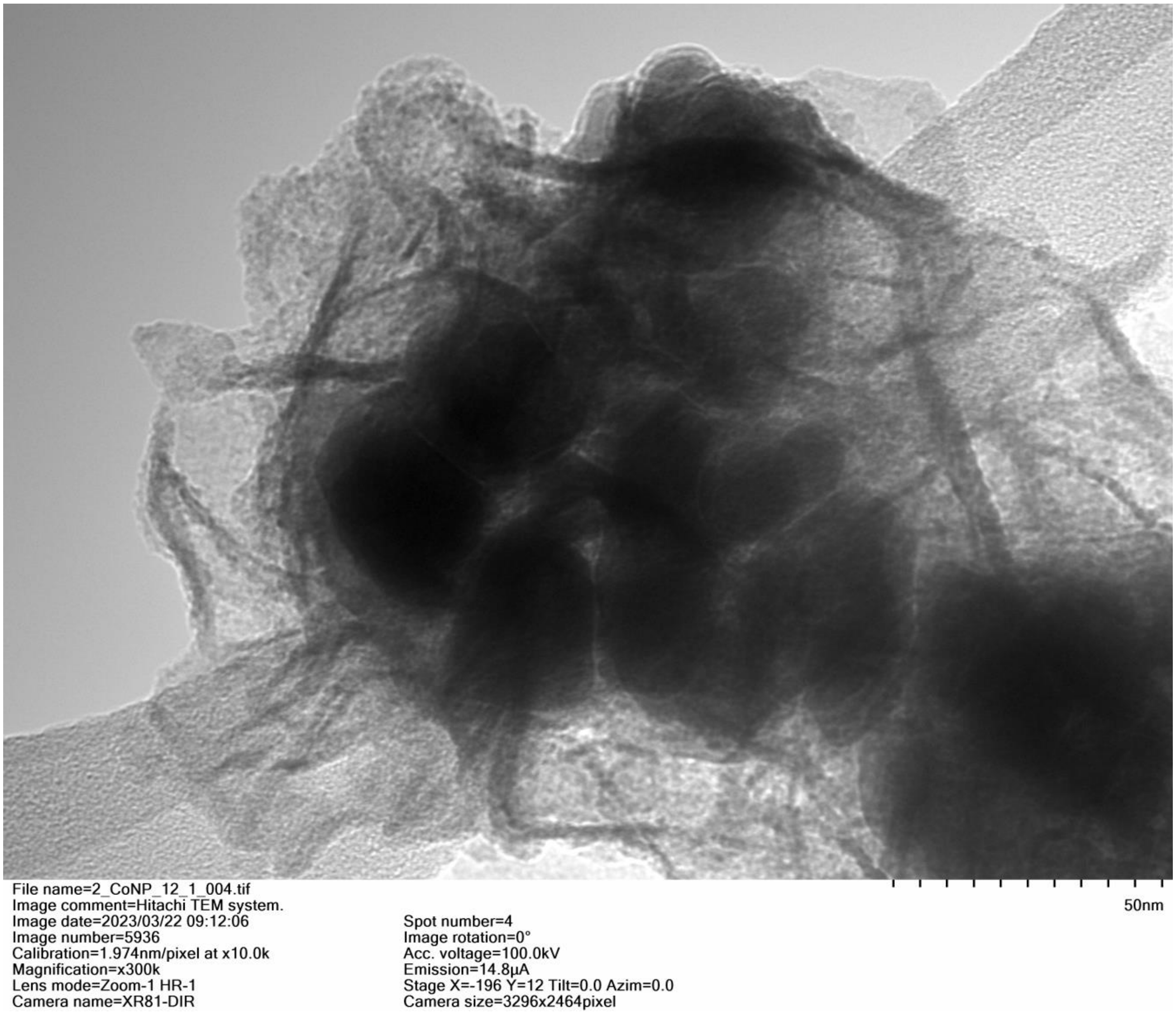

Figure S4. Tem image PEG/CoB-NP

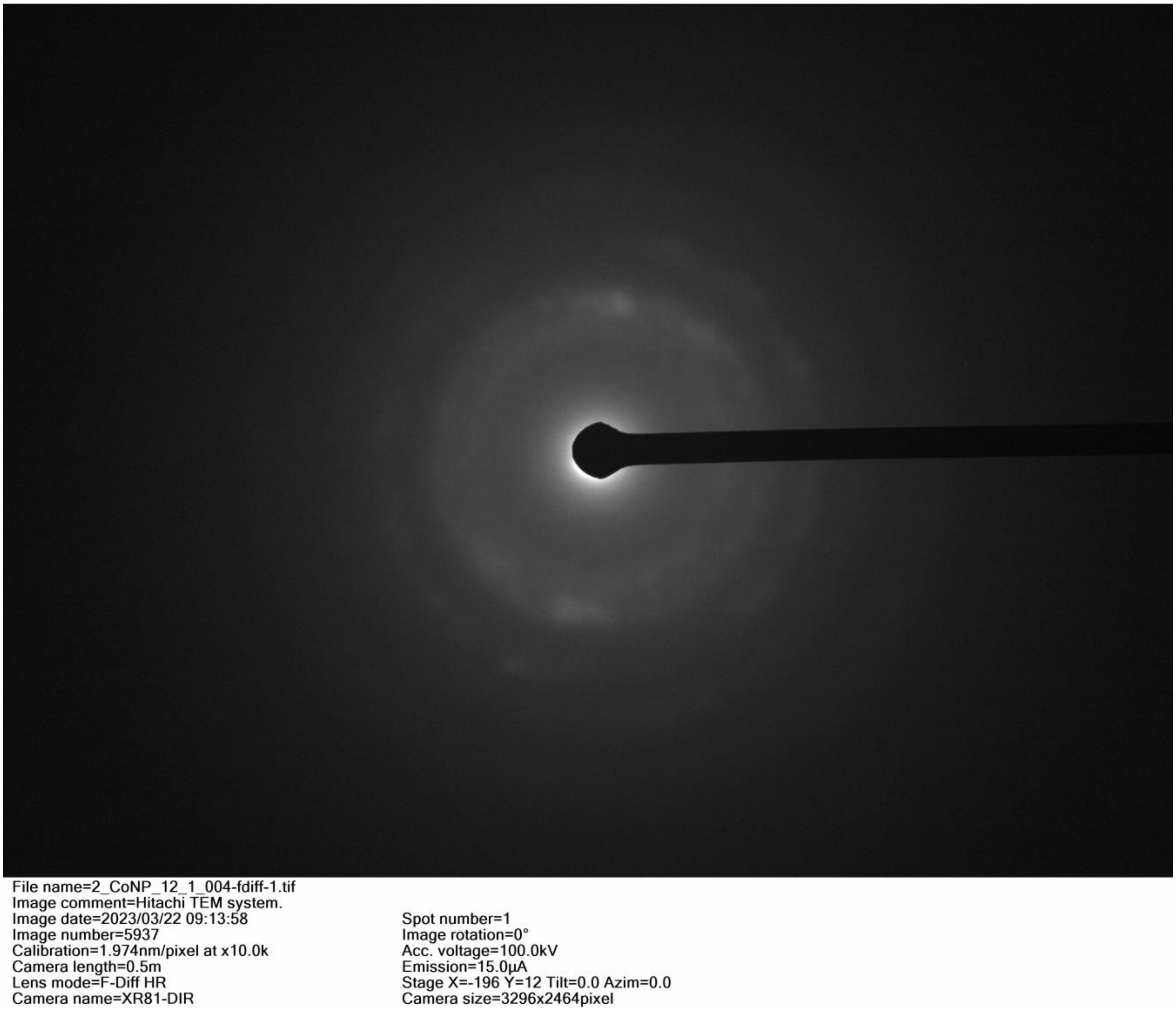

Figure S5. SAED image PEG/CoB-NP from figure S3

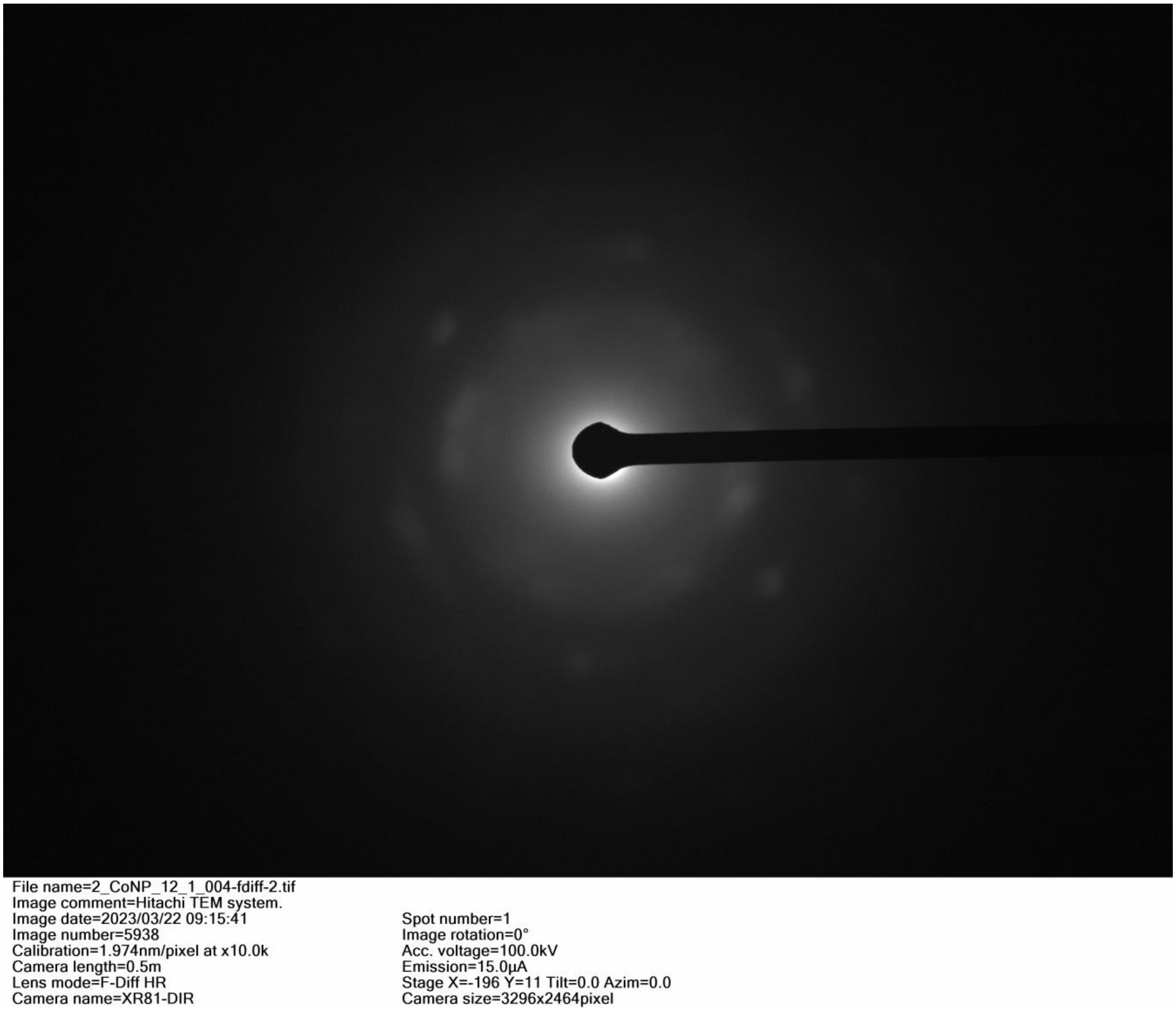

Figure S6. SAED image PEG/CoB-NP from figure S4

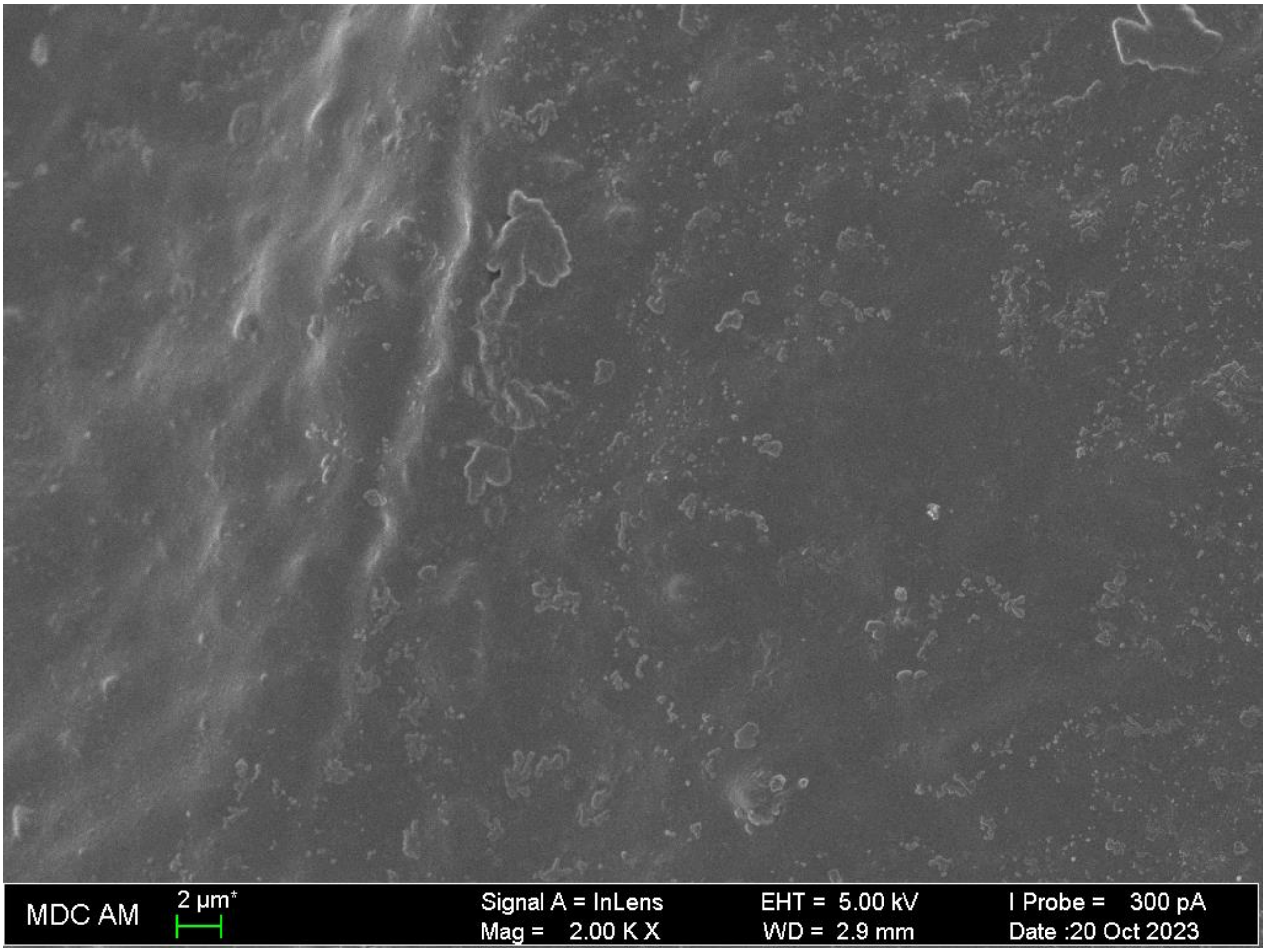

Figure S7. SEM image of ABS/[PEG/CoB-NP] with 0.3% nanoparticle loading

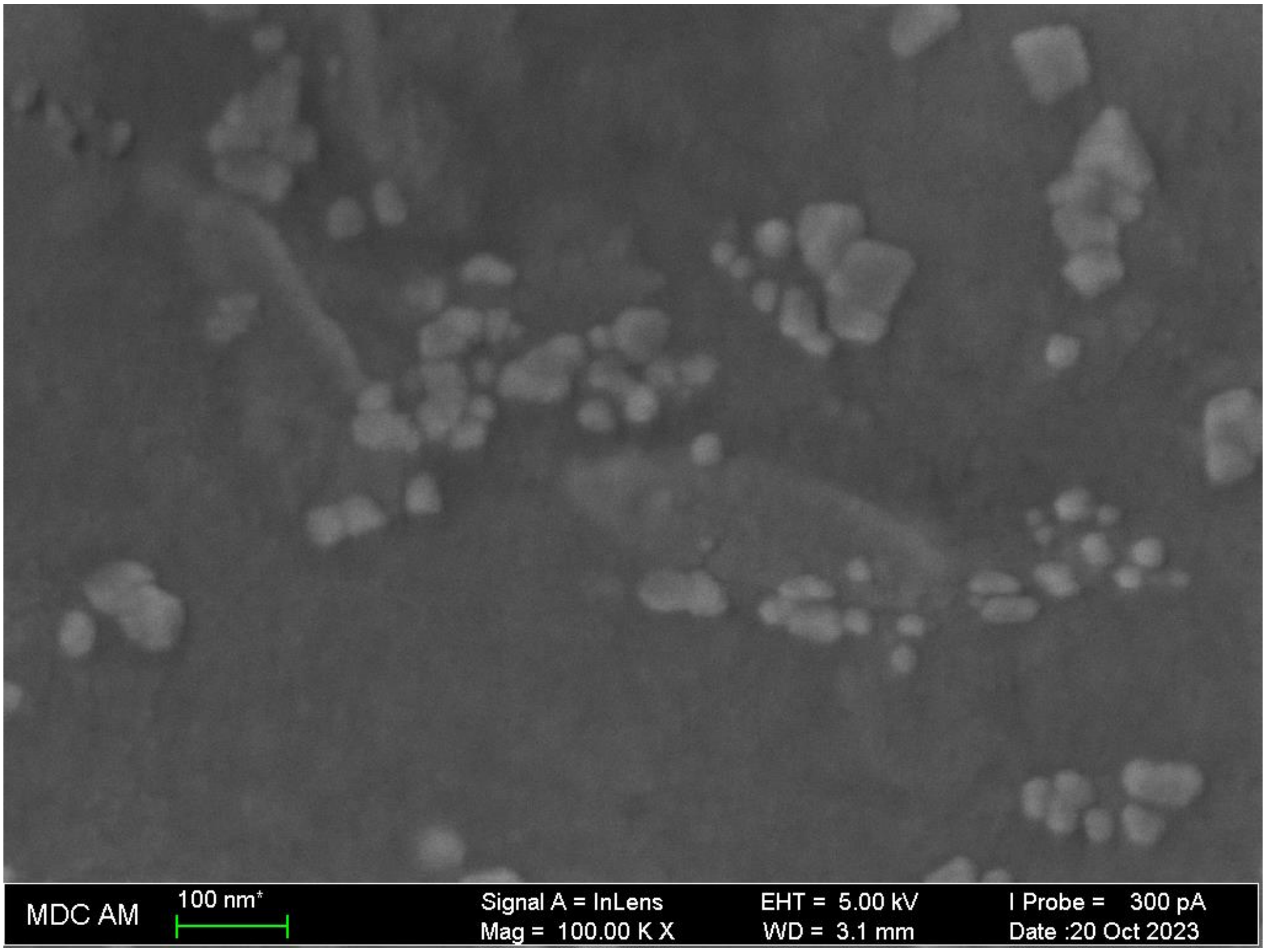

Figure S8. SEM image of ABS/[PEG/CoB-NP] with 0.3% nanoparticle loading

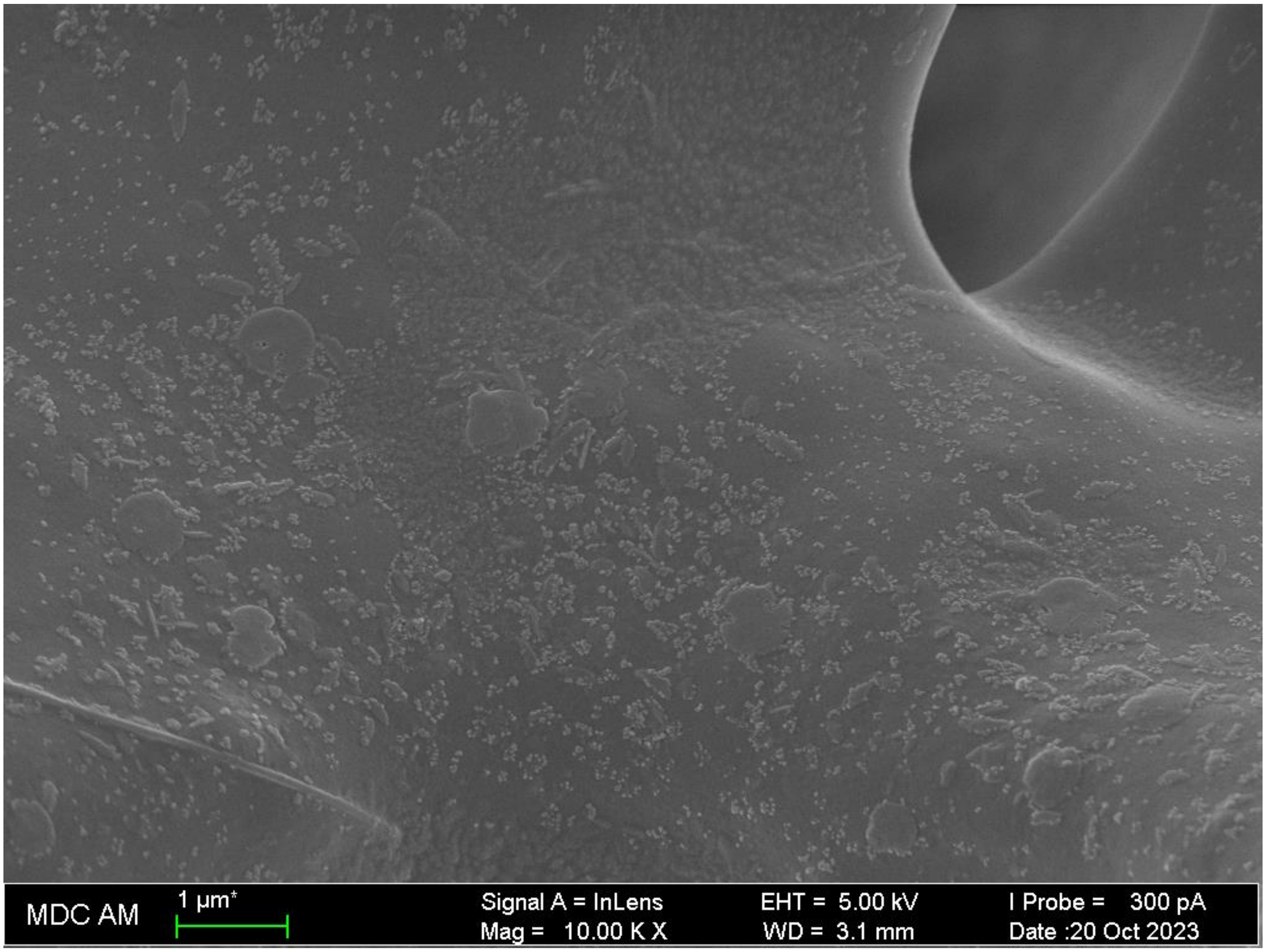

Figure S9. SEM image of ABS/[PEG/CoB-NP] with 1.5% nanoparticle loading

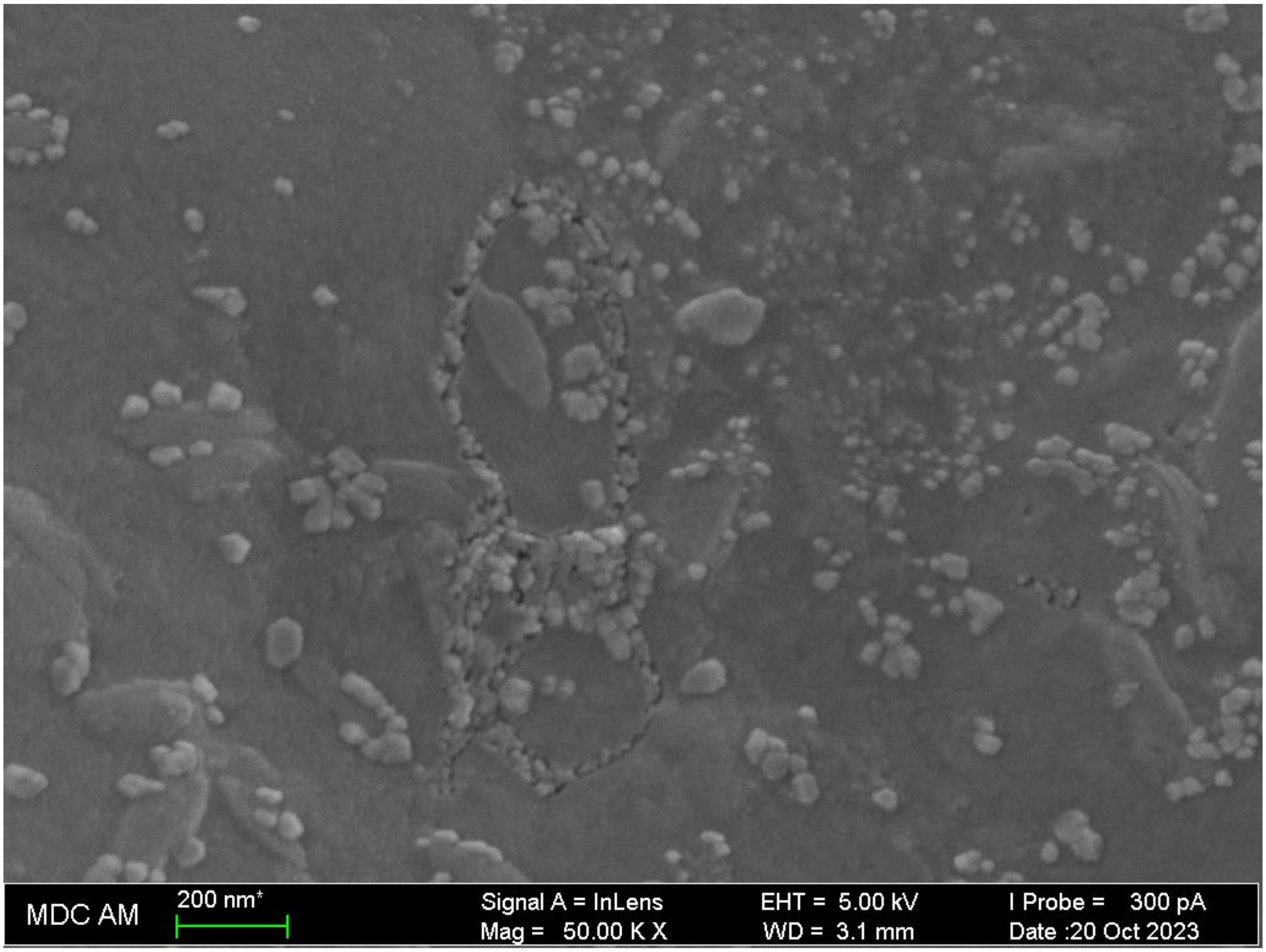

Figure S10. SEM image of ABS/[PEG/CoB-NP] with 1.5% nanoparticle loading

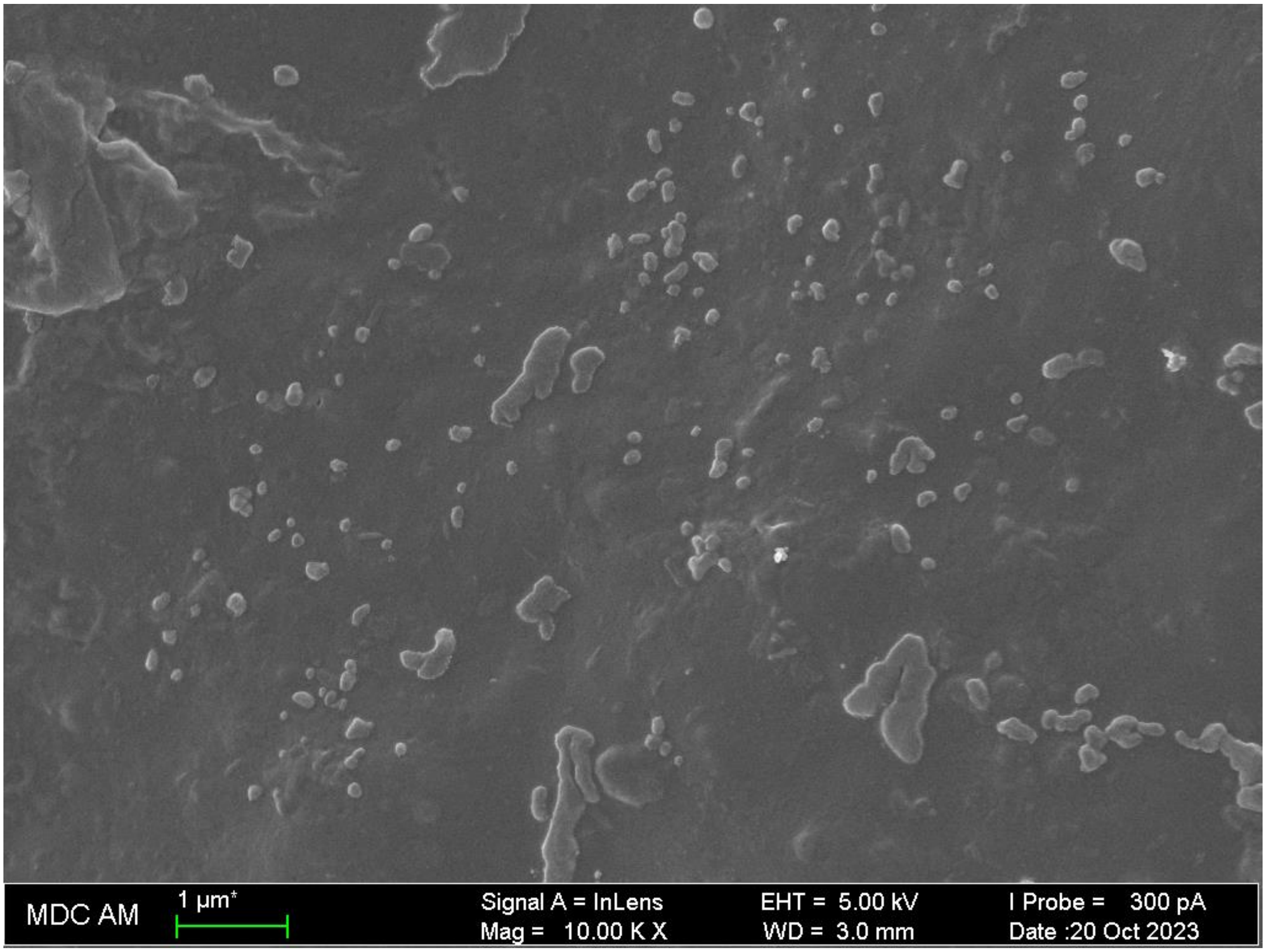

Figure S11. SEM image of ABS/[PEG/CoB-NP] with 3% nanoparticle loading

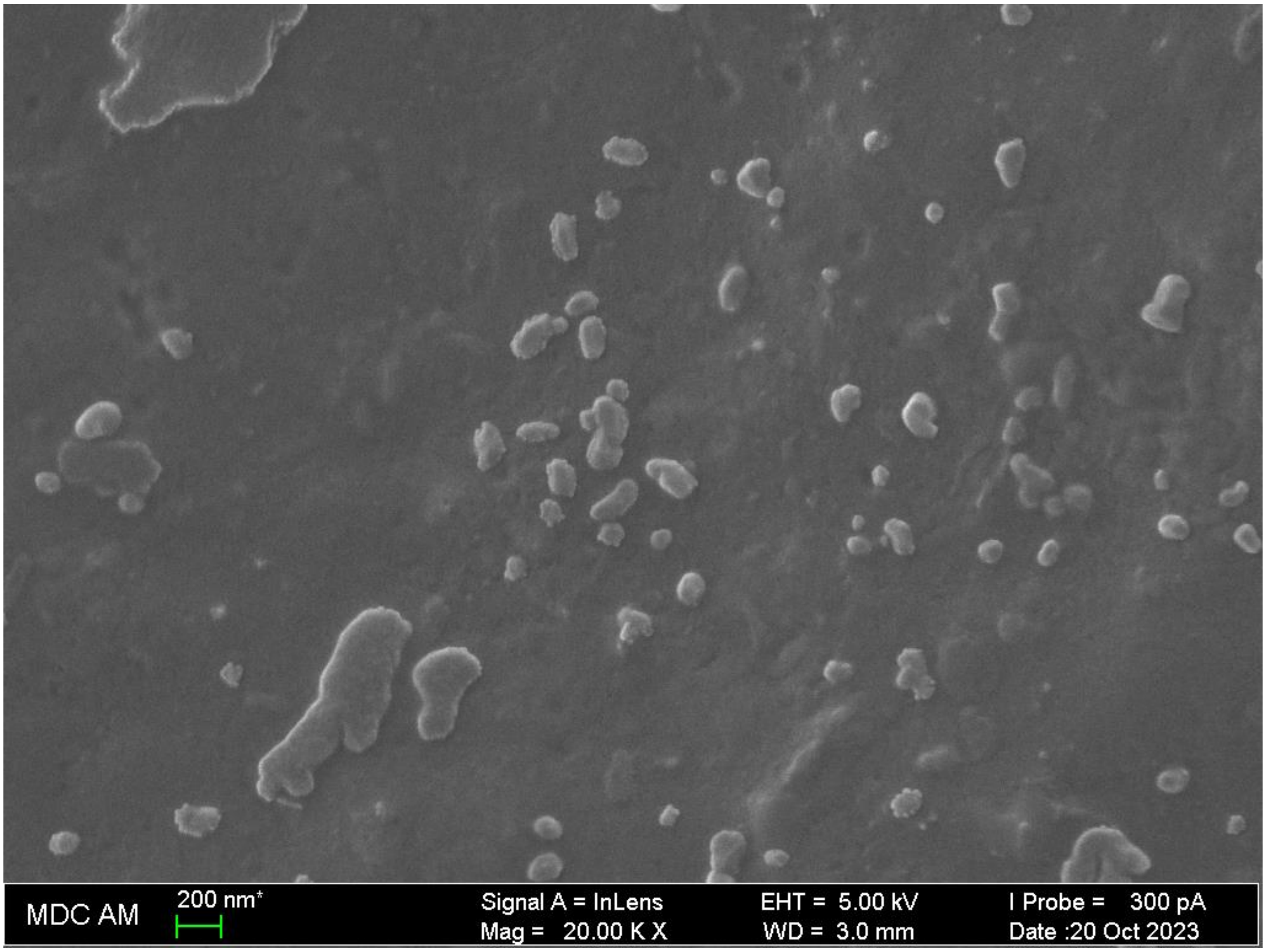

Figure S12. SEM image of ABS/[PEG/CoB-NP] with 3% nanoparticle loading

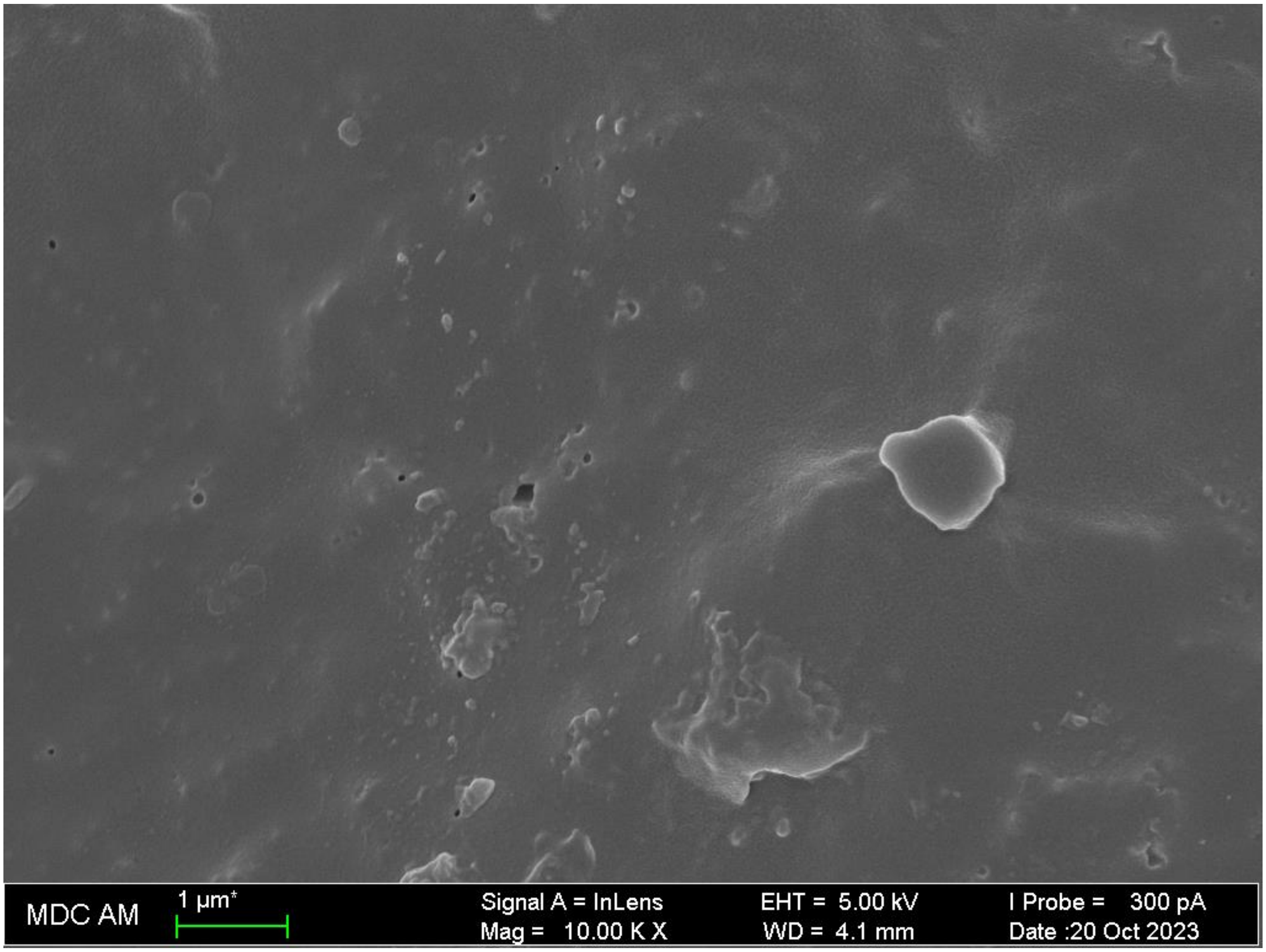

Figure S13. SEM image of ABS/[PEG/CoB-NP] with 6% nanoparticle loading

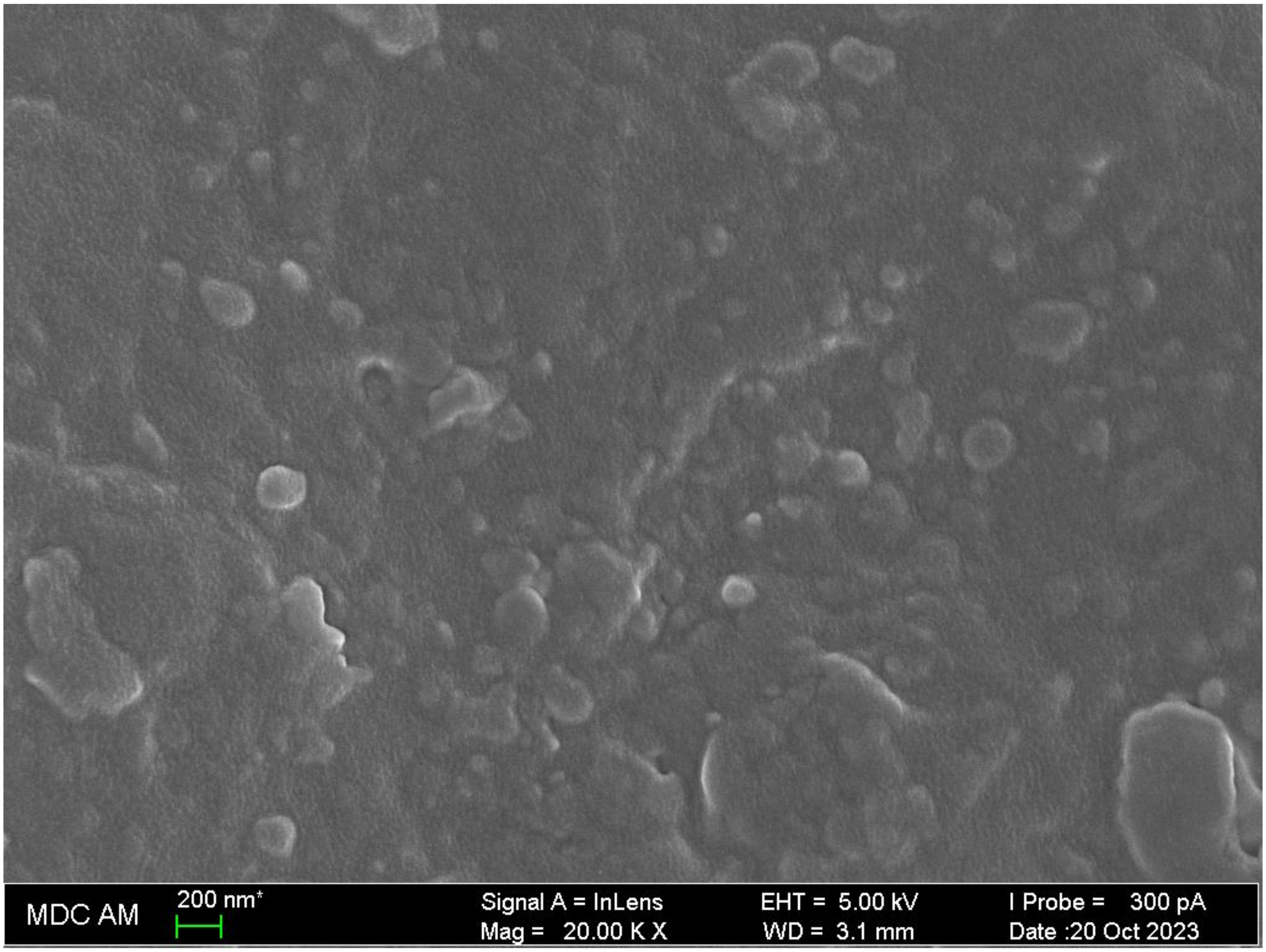

Figure S14. SEM image of ABS/[PEG/CoB-NP] with 6% nanoparticle loading
